# Supplementary material for: A High-Performance Antibacterial Nanostructured ZnO Microfluidic Device for Controlled Bacterial Lysis and DNA Release
Source: Antibiotics (Basel). 2023 Aug 2;12(8):1276. doi: 10.3390/antibiotics12081276 (PMC10451374; doi:10.3390/antibiotics12081276)
Supplement: Supplementary file 1 [file antibiotics-12-01276-s001.zip › antibiotics-2480414-supplementary.pdf]

A High-Performance Antibacterial Nanostructured ZnO Microfluidic Device for Controlled Bacterial Lysis and DNA Release

Yvonni Xesfyngi [1], Maria Georgoutsou-Spyridonos [1], Abinash Tripathy [2], Athanasios Milionis [2], Dimos Poulikakos [2], Dimitrios C. Mastellos [3], and Angeliki Tserepi [1,*]

[1] Institute of Nanoscience and Nanotechnology, National Center for Scientific Research (NCSR) "Demokritos", Patr. Gregoriou E' and 27 Neapoleos Str., 15341 Aghia Paraskevi, Greece; yvonnicx@gmail.com (Y.X.); maria.georgoutsou.spyridonos@gmail.com (M.G.-S.)
[2] Laboratory of Thermodynamics in Emerging Technologies, Department of Mechanical and Process Engineering, ETH Zurich, 8092 Zurich, Switzerland; atripathy8@gmail.com (A.T.); athanasios.milionis@ltnt.iet.mavt.ethz.ch (A.M.); dpoulikakos@ethz.ch (D.P.)
[3] Institute of Nuclear & Radiological Sciences and Technology, Energy & Safety, National Center for Scientific Research (NCSR) "Demokritos", Patr. Gregoriou E' and 27 Neapoleos Str., 15341 Aghia Paraskevi, Greece; mastellos@rrp.demokritos.gr
* Correspondence: a.tserepi@inn.demokritos.gr

Supplementary Material

EtBr was intercalated to *E. coli* DNA in amounts ranging from 0.1 to 1 ng, and a gel image was obtained from which the fluorescence intensity (FI) was estimated via ImageJ. Figure S1 shows FI as a function of the *E. coli* DNA amount, where a rather good linear dependence is indicated for DNA up to 1 ng (and FI up to 5000 a.u.).

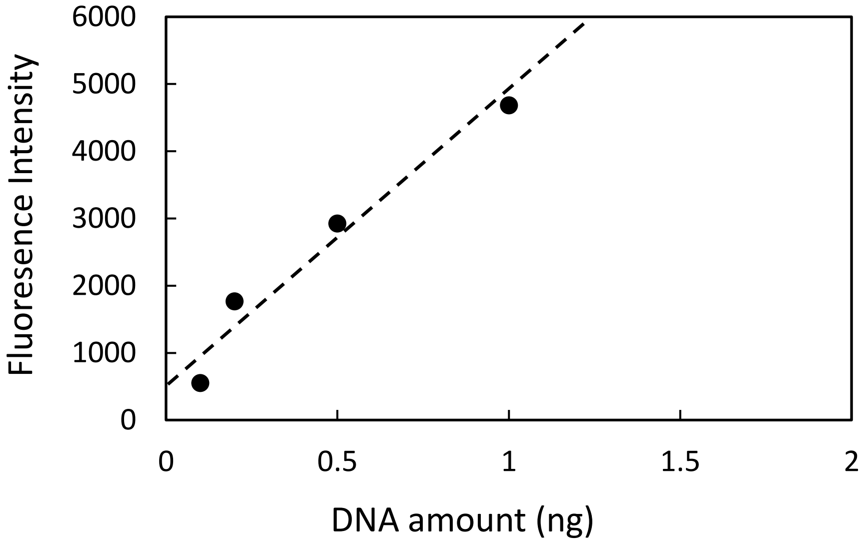

Figure S1. Standard curve for fluorescence intensity as a function of *E. coli* DNA in the range 0.1–1 ng.
